# “*It Is as if I Gave a Gift to Myself*”: A Qualitative Phenomenological Study on Working Adults’ Leisure Meaning, Experiences, and Participation

**DOI:** 10.3390/bs14090833

**Published:** 2024-09-18

**Authors:** Kubra Sahadet Sezer, Esra Aki

**Affiliations:** 1 Division of Occupational Therapy, Brunel University London, London UB8 3PH, UK; 2 Graduate School of Health Sciences, Hacettepe University, Ankara 06100, Türkiye; 3 Division of Occupational Therapy, Faculty of Health Sciences, Hacettepe University, Ankara 06100, Türkiye; esraaki@hacettepe.edu.tr

**Keywords:** leisure, leisure participation, adults, well-being, meaning of leisure, occupational justice

## Abstract

Leisure participation is a fundamental human and occupational right throughout life for working people, particularly in adulthood. A total of 28 working adults representing diverse regions of Turkey, from middle-class backgrounds, aged between 25 and 50, and without any known health conditions, were interviewed to gain insights into their leisure participation during the period September 2021–May 2022. The acquired data were analysed using the interpretative phenomenological analysis (IPA) approach. The analysis identified six main themes and twenty-two subthemes: the meaning of leisure, recovery from work, facilitators and barriers, well-being, occupational injustice, and flow of life. Participants distinguished between “free time” and “leisure time”, defining the latter as purposeful engagement in enjoyable, meaningful activities. This study emphasises the dynamic interplay of factors influencing leisure participation among Turkish working adults, including working conditions, financial resources, social support systems, and opportunities for participation, with some effects of COVID-19 pandemic. One can shift from well-being to a lack of well-being, and this can result in occupational injustices that may arise in the flow of life, as unsupportive consequences of participation limitations among working adults. By acknowledging and enhancing leisure as a crucial aspect of well-being, this research underscores the importance of promoting resilience and holistic health among working individuals.

## 1. Introduction

### 1.1. Leisure Definition

The term “leisure” is ubiquitous in daily life, yet there is no single definition but rather several characterisation attempts. Leisure is a significant occupational life domain according to the Occupational Therapy Practice Framework [1] and is described as a non-obligatory activity that is truly driven and carried out during free time, that is, when there is no commitment to participate in employment, self-care, or sleep [2]. Based on economic, psychological, and sociological literature, leisure has four categories: leisure as time-use, leisure as household consumption and expenditures, leisure as a lifestyle choice, and leisure as an experience [3] (pp. 633–658). In addition, leisure activities take place outside of regular work hours; they are deeply rooted in the culture, and individuals are free to choose whether or not they will engage in or participate in the activities [4,5].

### 1.2. Leisure Participation and Meaning

Participating in activities that are perceived as meaningful, along with belief in the meaningfulness of one’s life, contributes significantly to an individual’s well-being [6,7]. Leisure participation refers to a person’s involvement in leisure activities, meeting their physiological, psychological, and social needs [8]. Vital engagement, which means being part of long-lasting, enjoyable, and meaningful relationships, is seen as an important part of healthy development and is linked to meaningful leisure activities [9]. The enduring significance of leisure in fostering meaningful engagement with life is that leisure activities across various cultural domains provide ample opportunities for engagement and meaning-making [10,11]. Iwasaki describes how leisure participation contributes to meaning-making within specific cultural contexts, and the latter explores “what and how people define or perceive the meaning of leisure [12,13]. 

### 1.3. Leisure in Working Adults

It is known that leisure time is usually referred to as non-working time; thus, research on leisure engagement for working adults has been studied widely in organisational behaviour. Sonnentag discussed the recovery theory with respect to working adults and emphasised the experience approach. This approach is signified by the psychological states that individuals experience during their non-working time. It encompasses how they live and perceive their non-working time, including psychological detachment from work, relaxation, mastery, and control [14]. Similarly, Newman et al. discussed leisure from the standpoint of experience and meaning rather than leisure activity types and frequency [4] (pp. 633–658). Thus, to have a complete understanding of participation, it is essential to have a comprehension of the subjective experiences associated with leisure [15].

### 1.4. Leisure and Well-Being

The current trend of leisure participation implies positive experiences among adults, such as improved physical health and psychological well-being [16]. Similarly, evidence demonstrates the significant role leisure participation has in fostering positive social interactions and developing intimate relationships [17]. The findings of Elsden et al. [18] suggest that more engagement in activities is linked with better general health and vitality. Li et al. [19] emphasised the importance of leisure activities in maintaining the well-being of elderly adults. Satisfaction with leisure activities can act as a bridge between a person’s sense of well-being and experiences of happiness. For instance, a study conducted by Argan et al. [20] suggested a significant effect of satisfaction in moderating processes between experiences and happiness. In a recent scoping review that highlighted ongoing discourse surrounding the conceptualisation of leisure, dealing with leisure and well-being served as an important reminder of the significance of extensively evaluating the complex and multifaceted role of leisure in individuals’ everyday lives [21]. However, it is well known that well-being and leisure are connected in a way that works both ways. Leisure activities make people happier, and their happiness affects their desire or ability to engage in leisure activities [22]. A systematic review conducted by Gramabi et al. [23] suggested significant education, disability (individual factors), and social networks with family, friends, etc., (environmental factors) as significant determinants of social and leisure participation among individuals. Moreover, Sardina et al. [24] identified the lack of available activities, lack of awareness regarding activities, limited social networks, and transportation as the most common leisure barriers among older adults residing in subsidised housing. A comparative study explored barriers associated with leisure participation among adults with and without disabilities. According to the study findings, poor health and injury were the most frequently cited barriers to participation for adults with disabilities, while lack of time and too many other commitments were often cited among adults without disabilities [25]. Another study demonstrated that adults experiencing poverty prefer to participate in low-cost and free leisure activities but lack access to opportunities, accommodation, and assistance that the general population enjoys [26].

### 1.5. Flow and Leisure 

According to Csikszentmihalyi, flow is a psychological state characterised by complete immersion and focused attention in the present moment [27]. It is often regarded as a crucial element that contributes to the sense of fulfilment and significance individuals derive from engaging in leisure activities. More particularly, a feeling of exhilaration and a deep-seated sense of enjoyment and fulfilment are achieved while actively engaged in the activities rather than during relaxing or passive moments [28]. This indicates that flow is experienced during leisure activities that involve substantial effort and the proactive attitude of the individuals [27]. Studies also show that over the course of a person’s life, this immersive state is not static; rather, it changes to accommodate the evolving demands, passions, and developmental stages. Various elements, such as sociocultural frameworks, psychobiological changes, and psychological traits like openness to experience can influence the stability of flow experiences throughout adulthood [29]. When people reach adulthood, their pursuit of flow experiences could change to focus on schooling, establishing personal and professional identities, and career-related activities. In the midlife years, flow can be associated with the benefits and challenges of balancing multiple roles [30]. Moreover, the experiences of individuals can be influenced by significant life transitions and transformations in roles that occur during adulthood, such as getting married, becoming a parent, and retiring. Additionally, functional declines with age create a discrepancy between challenges and skills, which can impact the continuity of flow experiences in adulthood [31].

### 1.6. Leisure as a Right and Occupational Justice 

As stated in the Universal Declaration of Human Rights, “each person has the right to rest and leisure, which include a reasonable limit on hours of work and paid vacations at regular intervals,” [32] yet it has been far too long overlooked [33]. Occupational science advocates that occupation is considered as essential as air, food, and water for humans [34]; it can be inferred that humans and societies would progress towards a fairer world by expanding the concept of human rights to encompass participation in various occupations [35]. Occupational justice from the occupational science perspective is founded on the notion that participation in an occupation has the capacity to impact one’s health and has six different aspects: occupational imbalance, occupational deprivation, occupational disruption, occupational alienation, occupational marginalisation, and occupational apartheid [36]. Four key concepts of occupational justice are highlighted to understand how participation in occupations impacts health and well-being. Occupational disruption involves temporary interruptions in occupational engagement due to events such as illness or life transitions. Occupational deprivation refers to situations where external factors prevent a person from engaging in meaningful occupations, such as geographic isolation or economic constraints. Occupational alienation happens when people engage in occupations that they find meaningless or unfulfilling, often due to societal pressures or constraints. Occupational imbalance occurs when there is an unequal distribution of time spent on different types of activities, leading to stress or lack of fulfilment [34]. The occupational justice framework includes participation; this point is also in line with the idea that everyone has the right to a wide range of meaningful occupations that meet their specific needs and help them grow as a person [2]. Within the scholarly literature, there is an understanding that limitations to meaningful occupation might be viewed as experiences of injustice [35].

### 1.7. Objective of the Study

Leisure in occupational therapy and occupational science with adults has been explored in several studies, mostly focused on people with disabilities, such as adults with Multiple Sclerosis [37] or vulnerable adults like those who have poverty [26] or older adults (aged 60 and over) participating in community-based creative arts, which reported many benefits, including improved occupational performance and positive well-being outcomes [38]. However, there is a scarcity of resources focusing on leisure participation among working adults from an occupational therapy perspective. The present investigation seeks to bridge this gap by delving into the leisure participation of Turkish working adults and its meaning to them. Exploring the role of leisure among working adults can provide insights into the ways people balance work-related engagements with their leisure activities, significantly influencing their mental health and quality of life. Thus, the present study aims to offer valuable insights that could contribute to the practices of occupational therapists in optimising the experiences of working adults by enhancing positivity, growth, and a meaningful life.

## 2. Materials and Methods

### 2.1. Study Design

An exploratory phenomenological approach was employed which places emphasis on subjective and experiential dimensions of human existence and explores individual perspectives and real-life encounters of participants in their environment [39,40]. The interpretative phenomenological approach was utilised, which involves the interpretation and understanding of data via an integrative process between the researcher and the subject being studied [41]. Thus, IPA facilitates the construction of insightful interpretative accounts of real-life subjective experiences [42].

### 2.2. Sampling and Participant Recruitment

**Figure 1:**
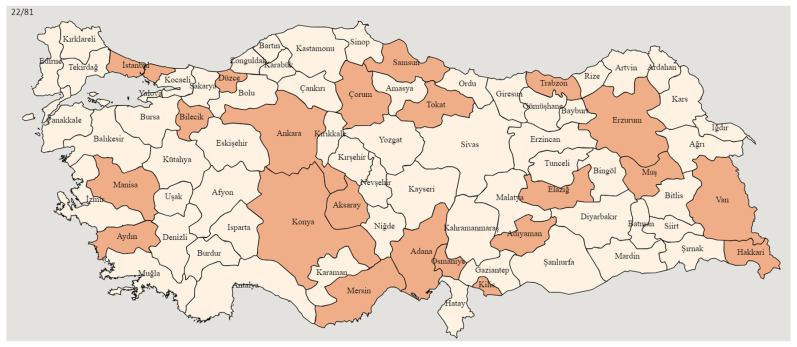
Turkey map with 81 provinces. Orange ones represents the provinces of the participants.

A total of 28 working adults who live in Turkey from middle-class backgrounds, aged between 25 and 50, and without any known health conditions were selected using the purposive sampling technique and the snowball sampling technique [43]. The combination of purposive and snowball techniques [44] resulted in a well-rounded and diverse participant cohort which enriched the study’s findings and contributed to a more in-depth exploration of the research topic. For the purpose of participant recruitment, recruitment criteria were initially shared on social media platforms such as Instagram and larger WhatsApp groups. Initially, a purposive sampling approach was used to select best-fit participants, ensuring the deliberate selection of participants who possessed the experiences necessary for gaining deep insights into the topic [45]. Following the initial recruitment process, a snowball sampling approach was employed for participants from various regions across Turkey (See Figure 1: Turkey Map). Participants from the initial purposive sample were asked to refer other Turkish working adults who could provide valuable insights to the study. The snowball sampling process was carried out until data saturation was achieved [46]. Those expressing voluntary interest were contacted, and the study procedures were explicated via Zoom video conferences.

### 2.3. Instrument

The occupational performance assessment of play and leisure was measured by a self-developed semi-structured interview guide (see Table 1). To create a person-based occupational profile, the assessment should focus on the evaluation of play and leisure activity (i.e., activity interest and activity choice) and the evaluation of play and leisure experiences (i.e., subjective experience, context of the activity, personal meaning, and satisfaction with experience). Thus, this semi-structured interview guide focused on six key areas: (1) activity interest considers activity preferences; (2) activity choice focuses on what, done with whom, and how often; (3) subjective experience highlights freedom of choice, internal motivation, active participation, and irrational choices; (4) context of the activity focuses on cultural, social, personal, spiritual, temporal, and virtual environment; (5) personal meaning demonstrates the satisfaction of conscious and unconscious needs; (6) satisfaction with experience captures overall satisfaction with the play and leisure activities [2] (See Table 1).

### 2.4. Data Collection

After obtaining ethical approval from the Istanbul Medipol University Non-Invasive Research Ethics Committee (Approval number: 2020/657), the data collection phase of this study transpired between September 2021 and May 2022. Alase [47] posits that in phenomenological research, the participant count can fluctuate between two and twenty-five. Nevertheless, in the present investigation, the sample size was determined as 28, a figure reached upon achieving data saturation. The participant recruitment process adhered rigorously to ethical considerations and safeguarded participant rights. Comprehensive information regarding the study’s objectives, purpose, expected time commitment, and responsibilities was provided to each prospective participant, fostering an environment of trust and rapport between researchers and participants. Prior to engaging in semi-structured interviews, explicit verbal and written consent was obtained from each participant, with all interviews being video-recorded upon participants’ consent. The interview duration spanned between 20 and 65 min, with an average of 33 min. The interview protocol comprised two distinct series, with the initial phase predominantly featuring demographic questions. These questions were strategically designed to cultivate a sense of trust and rapport between the interviewer and participants. Notably, data collection transpired from different regions of Turkiye and amidst the COVID-19 pandemic, necessitating the utilisation of video conferencing technology for semi-structured interviews. This approach facilitated unrestricted data collection, overcoming geographical, temporal, and physical constraints [48].

### 2.5. Data Analysis

Data were analysed using MAXQDA 2022 software (VERBI Manufacturer, Berlin, Germany). Interview videos totalling 1000 min were uploaded into the software, which were then transcribed into a Word document. The transcribed text underwent grammar and spelling checks, and errors were corrected. Later, a close reading of the transcripts was carried out. This initial step allowed immersion in the data, capturing the atmosphere and setting in which the interviews were conducted. Notes and reflections were made to capture important information, particularly focusing on the content, language use, and context. Additionally, distinctive phrases and emotional responses were also highlighted. An interpretative phenomenological analysis (IPA) approach was used to analyse the collected data. The detailed and comprehensive notes were transformed into emerging themes, aligning with a deductive approach that involved the use of predefined themes in the data.

An inductive approach with predefined codes and themes was first used. Then, open coding was used to find new codes, categories, and patterns in the data. We further refined our analysis after the completion of deductive and inductive coding. In some cases, predefined codes were renamed, such as the temporal dimension, which was renamed “flow of life”. Predetermined themes were compared with themes that emerged inductively. Later, connections between themes were observed and clustered together based on conceptual similarities and differences. Before identifying the connections, the themes were compiled from the whole transcript. Themes and subthemes were finalised with a strong evidential base. Thus, the use of the deductive approach helped in addressing research questions in a structured manner and with a theoretically grounded framework; the inductive approach allowed for the exploration of valuable insights and themes that emerged from the data itself [49]. According to Richards [50], the deductive and inductive “both are always involved, often simultaneously”, and “it is impossible to go theory-free into any study” [51]. In order to ensure reliability and enhance trustworthiness of the study findings, the second researcher independently reviewed the identified codes, themes, and concepts. Misunderstandings or errors identified during this process were addressed.

## 3. Results

### 3.1. Sociodemographic Data of Participants

This study has of a diverse group of 28 individuals, equally divided by gender, with 14 males and 14 females, aged between 25 and 50, with an average age of 34. The educational background varies, with the majority holding a bachelor’s degree (15 individuals), while others have completed 2 years of college (6 individuals) or pursued a master’s degree (5 individuals) or even a PhD (1 individual). Only one individual has completed 6 years of university study (in medicine).

Living arrangements are diverse, with most participants living with a family including children (13 individuals), followed by those living with a spouse or partner (6 individuals). The group’s monthly income ranges from below 1x the minimum wage in Turkey to over 4x, with most earning between 2x and 3x the minimum wage.

In terms of work, most are employed full-time (24 individuals), with some working part-time (4 individuals). Work hours vary, with the majority working 40 to 50 hours per week. A small portion also engages in night shifts or hybrid work setups (Table 2).

### 3.2. Qualitative Data Analysis Results

The IPA identified six main themes—the meaning of leisure, recovery from work, facilitators and barriers, well-being, occupational injustice, and flow of life—and twenty-two subthemes (See Table 3).

1. Meaning of leisure

The current study aimed to explore how Turkish society views leisure and free time. Based on a pilot study, participants were asked to define the concept of leisure. The terms “free time” and “leisure time” were used interchangeably in Turkish society, prompting us to investigate how participants perceived and differentiated these concepts.

1.a. Freedom

“Free time-boş zaman” in Turkish evokes a deep sense of freedom and emancipation for the participants. It is seen as a priceless chance to escape the burden of commitments and engage oneself in pursuits that bring joy and fulfilment. The Turkish term for leisure, “serbest zaman,” is semantically linked to freedom, evoking a sense of liberation from life’s responsibilities. Participants viewed free time as an opportunity to break free from obligations and engage in activities they desired, as stated below.

“*When I say free time, it makes people feel like I have an obligation and you’re getting rid of it. For example, use your free time as if you were under arrest and go out to the courtyard. We live with responsibility and anxiety, and when we feel happy, it feels like being free.*”

(Dilara, 26 yo, F, Psychologist, lives in Aksaray province)

Serkan (28 yo, M, Laboratory Technician, Samsun province) effectively summarised the meaning of personal freedom and quoted, *“I think when I can do whatever I want for myself, it makes me freer.”* This quotation emphasises the notion that leisure offers an opportunity for people to exercise autonomy and make decisions in line with their own preferences and aspirations. Moreover, Fatma (41 yo, F, Government Official, lives in Mersin Province) offered insightful information about their leisure strategy, as quoted,

“*I do my leisure on Sundays… I do it by putting them in order. I can’t do it due to busy weekdays. Now I have to plan, as I have very little time left. I do what I want; everything I do, I do willingly.*”

In conclusion, the participants’ understanding of “free time” was intimately tied to the concept of being released from life’s obligations, with a heavy focus on the ability to pursue individually gratifying activities. This viewpoint emphasises the function of “free time” in offering a much-needed break from everyday responsibilities and the chance to exercise autonomy in leisure pursuits.

1.b. Leisure time instead of free time

This subtheme clarified the difference between free time and leisure time. Participants offered a variety of perspectives on how they understood these ideas, highlighting the distinction between leisure time and free time based on the calibre of the time spent.

Distinguishing leisure time from free time was crucial for our study. All participants emphasised the distinction between leisure and free time, primarily related to the quality of time spent. Leisure was described as time individuals create for themselves, while free time was associated with empty or idle moments, such as scrolling on social media. As Gökçe (35 yo, F, Lecturer, lives in Istanbul province) stated, *“The concept of free time is analogous to something else in that it resembles time left over from work; however, during leisure time, we create more for ourselves.”*.

Turgut (30 yo, M, Doctor, lives in Konya province) also expressed similar thoughts. *“By “free (boş zaman in Turkish)”, I mean “empty”. My leisure activities, however, are not idle”*. In addition, Sude (26 yo, F, Speech Language Therapist, lives in Aydın province), for whom the concept of leisure was more common in society but semantically meant wasting time, said, *“Leisure is closer to me. Because free time evokes emptiness. As if it were a useless time. Actually, it shouldn’t be that way.”*

Based on the participant quotes, interest and predetermined time periods are the main forces behind leisure. It is worth noting that the time spent on social media, particularly activities like scrolling, was not considered a leisure activity by the participants. Ahmet (32 yo, M, Engineer, lives in Istanbul province) argued,

“*Free time is like a time period when I watch something meaningless on Netflix that will completely empty my mind.*”

One participant said that leisure is the idea that people are biological, psychological, and social beings whose tastes change over time and that work and productivity are not the only things that matter in life. They also said that time should be used in a way that fits your choices. Mustafa (48 yo, M, Teacher, lives in Elazığ province) quoted,

“*A human being has feelings and emotions; they are not merely like machines. He desires to use time in a different way. Some people do this through walking, while others do it through other activities, such as making art or having fun with their kids.*”

Nonetheless, participants indicated that leisure is a source of vitality and adds depth to life. Also, it is significant in shaping unique identities and experiences. They emphasised learning through leisure and its role in creating a fulfilling, distinctive life story. As Özge (30 yo, F, Research Assistant, lives in Ankara province) stated,

“*In my opinion, what distinguishes me from a departed person—what sets me apart from someone who has passed away—are my leisure activities… If I only come and go between work and home for the next 30 years, I would consider this to be a life I have never experienced. The process is analogous to story writing.*”

In conclusion, the subtheme emphasises the participants’ agreement on the distinction between leisure time and free time, with leisure characterised by active involvement, meaningful activities, and personal interests, while free time is associated with inactivity and emptiness. Overall, the participants stress the importance of leisure in sustaining life, forming identities, and creating a meaningful life story.

1.c. Me time

“Me time” is a phrase that, according to the respondents, places an emphasis on solitary or group activities that encourage rest and personal well-being. Similar to the concept of “me time,” another common expression of leisure by the participants is “time for themselves,” which involves individuals spending time alone and enjoying personal activities independently and without obligations. This period fosters relaxation, enjoyment, and quality time alone or with loved ones, emphasising breaks from responsibilities and prioritising personal well-being. Gökhan (45 yo, M, Journalist, lives in Adana province) argued,

“*When I think of leisure, I think of things where I can be alone with myself and do stuff with quality. You can do what you want to do, and it is your moments of pleasure that you set aside for yourself.*”

Moreover, participants in their 30s and older exhibited a stronger drive to seek leisure opportunities compared to younger participants in their 20s. Late young adults, aged 30 or older, exhibited a proactive approach to incorporating leisure into their lives, demonstrating both planning and efficient time management skills. According to participants, a crucial aspect of engaging in leisure is making time for it, which can also serve as a motivating factor for participation. As quoted,

“*I give up my sleep in the morning. I’ll go to work early and make myself coffee. I motivate myself there. No matter how busy you are, you can always find a few minutes to yourself.*”

Neriman (44 yo, Government Official, F, lives in Istanbul province)

In a nutshell, “time for themselves” or “me time” is a common expression of leisure that involves individuals dedicating time to enjoy personal activities independently and without obligations. Older individuals show a deeper dedication to leisure and better time management, which is an intriguing generational difference. Making time specifically for leisure is a motivational factor that emphasises the importance of leisure in their daily schedules.

2. Recovery from work

This theme specifies a break from work. Recovery from work is the process by which people regain and revitalise their physical, mental, and emotional well-being after exerting effort and coping with responsibilities at work or on the job. Maintaining overall well-being and a healthy work–life balance depends on it.

2.a. Relaxation

The subtheme emphasises how people view leisure as a vital means for relaxation, giving them a chance to escape their regular routines and find peace. Participants stressed the value of finding personal comfort and relief while having fun, which is frequently accomplished through pursuits that promote both physical and spiritual relaxation.

As stated by Dilara (26 yo, F, Psychologist, lives in Aksaray province) *“I feel comfortable when I do something I want”*, whereas Gökçe (35 yo, F, Lecturer, lives in Istanbul province) highlighted that engaging in activities outside their regular routine is a major source of relief. As stated,

“*I am very relaxed (took a deep breath and exhaled). I mean, when I do something outside of work and outside of the normal routine, if we go out, I feel such a relief.*”

Overall, this subtheme emphasises the importance of leisure in rejuvenation and the pursuit of activities that are different from one’s typical routine to feel liberated and neutralised from the stresses of daily life.

2.b. Mastery

This subtheme emphasises how common it is for individuals to want to learn new things and develop themselves, whether it is for their own personal or professional growth. Finding a skill to acquire was a common theme among participants, whether they were pursuing personal or professional development. The majority of respondents mentioned “self-development” both in the workplace and in their leisure time, which is defined in the leisure literature as the continuation of work. They preferred using their time to master topics in order to thrive.

“*I define leisure as things you can do to improve yourself.*

(Ozan, 31 yo, M, Engineer, lives in Istanbul province)

“*Leisure is also considered the activity that people do to renew themselves and complete their personal development.*”

(Ahmet, 31 yo, M, Engineer, lives in Düzce province)

In summary, this subtheme highlights how willing the participants are to use leisure as a platform for learning new things, expanding their knowledge, and fostering both personal and professional development. It emphasises how important leisure is for encouraging lifelong learning and ongoing self-improvement.

2.c. Detachment

This subtheme emphasises the importance of leisure in reducing the mental and physical fatigue brought on by the stress and pressures of the workplace. Participants experiencing mental and physical exhaustion from work aimed to alleviate fatigue, shed work-related burdens, and reduce psychological stress. Leisure participation, particularly on weekends, was effective in alleviating mental fatigue linked to work. For some, seeking leisure as a means of relaxation was synonymous with pursuing happiness. They acknowledged that while these activities might demand a significant amount of time, the benefits in terms of mental and physical well-being make the investment worthwhile. This is depicted in the quotes of participants mentioned below.

“*I feel mentally relaxed with that. If I do something on the weekend and forget what I did on Friday, I am happy to try to remember it on Monday morning. I try to provide mental relaxation.*

(Ali, 37 yo, Engineer, lives in Istanbul province)

“*I think of leisure as the time when people can relax. But this rest should include not only physical but also mental rest. Resting is actually being able to calm down for me.*”

(Sude, Speech and Language Therapist, 26 yo, F, lives in Aydın province)

While participants stated that leisure participation equates to relaxation, specifically as a means to detach from work and temporarily forget about work-related tasks, the results also found a contradiction between their initial definition of leisure time and their actual leisure activities. The literature typically defines leisure time as time not associated with work; some participants’ definitions contrast this notion. As Ali stated, *“So, we actually call it leisure, but it is intertwined at some points.”* This quote reiterates that the participants’ leisure activities and professional pursuits were interrelated.

To conclude, according to participants, leisure promotes better physical and mental health. The subtheme draws attention to a discrepancy between the participants’ original definitions of leisure time and their actual leisure pursuits, highlighting the intricate relationship between work and leisure in their lives.

3. Facilitators and Barriers

The third theme found in the data explained the facilitators and barriers to leisure participation. These included working conditions, financial resources, accessibility, roles and responsibilities, social support systems, and opportunities and some leisure activities not being financially accessible to everyone.

3.a. Working conditions

This subtheme highlights the considerable influence that working circumstances and schedules had on participants’ capacity to maintain a balance between work and leisure. According to the findings of this study, participants with flexible work hours found it easier to balance work and leisure, enhancing leisure-time participation. In Turkey, employees who work in non-governmental clinics or companies have 14 days annual leave in one year up to five working years in the same company. Some participants, mostly full-time employees with additional obligations outside of work, claimed that their working conditions had a negative impact on their participation in leisure activities. Despite working hard and looking for physical relaxation to relieve exhaustion, many voiced dissatisfactions about their inability to find time for the things they wanted to do.

“*We can say that it is a work-related problem, because if I had a few more days of annual leave, I could go to Eskişehir province (her hometown) to visit my family and friends. When I can’t participate in my leisure, that bothers me the most. There is really limited time after work. Sometimes I feel so bad when I can’t do the things I want to do. There are times when I even get sleepy and postpone going to sleep for the sake of leisure time*”

(Sude, Speech and Language Therapist, 26 yo, F, lives in Aydın province)

Overall, these results highlight the complicated relationship between work and leisure in people’s lives. Flexible work schedules can increase involvement in leisure activities, but the demands of a full-time job and additional obligations might present difficulties, such as exhaustion and a lack of free time. This emphasises the need to take into account work circumstances and schedules when looking into measures to encourage a good work–life balance and fulfilling leisure activities.

3.b. Financial resources

This subtheme demonstrates that economic limitations can present occasional barriers to participants’ engagement in certain leisure activities, particularly those that involve expenses such as travel. According to some participants, economic limitations hindered their ability to engage in certain activities, including travel. 

Similar thoughts were shared by Arif (40 yo, Security Officer, M, lives in Osmaniye province) as well. “*What is it that prevents me from doing what I like? I say money. Sometimes I can’t do them for economic reasons. These activities are also based on finance. It hinders you financially. You buy fuel to go somewhere.”*

To conclude, the respondents emphasised the impact of financial limitations on people’s leisure activities and choices. In order to think about strategies to make leisure more accessible and inclusive for people from various financial situations, it is crucial to understand these limitations.

3.c. Social Support (Spouse, Family Members, Friends)

The subtheme demonstrates how important social support is in influencing participants’ leisure activities. The most frequently expressed source of social support was the support of the spouse for married people and the support of the family for people living with their family. Some female participants revealed that sharing roles and duties with their spouses gives them free time and space to do what they want. As quoted,

“*My husband is my biggest facilitator for my leisure time and my life too.*”

(Fatma, 41 yo, Government Official, F, lives in Mersin province)

“*It makes easier to have an understanding partner. The circle of friends makes it easier.*”

(Orhan, 38 yo, Teacher, M, lives in Muş province)

While some participants discussed how the support provided by their colleagues facilitated their participation in leisure activities, other participants believed that the age and marital status of their colleagues affected their participation in leisure activities. As Erhan (Nurse, 28 yo, M, lives in a small county of the Manisa province) quoted,

“*I also have colleagues who are much older than me. It already creates a generational problem with them. Your expectations and wishes are different. Other than that, I am the only one who is single; everyone is married. That’s why nothing happens. People are constantly involved in their own plans.*”

The importance of social support in facilitating and enhancing leisure activities is emphasised by these studies as a whole. While co-worker support can improve the social components of leisure involvement, spouse and family support is essential in establishing the space and understanding for leisure. The impact of age and marital status on the dynamics of leisure time demonstrates the complexity of social interactions in leisure settings.

3.d. Accessibility

This subtheme underlines the importance of accessibility variables in affecting participants’ leisure possibilities and experiences. Accessibility refers to the tools or factors needed to access leisure activities, facilities, or time, such as the distance to a destination and/or the availability of conveyance. Participants noted that their province’s convenience enhanced leisure access, enabling them to allocate more time for leisure activities. Sahin (34 yo, Basketball Coach, M, lives in Kilis province) affirmed,

“*It is close, which makes my leisure time easier… I also have a bicycle. I reach there in ten minutes. As a facilitator, it leaves time for what I will do.*”

Conversely, living in a metropolitan area also brings challenges. Ali (37 yo, Engineer, M, lives in Istanbul province) expressed, “*Since we live in Istanbul, even when you go from one place to another, one hour passes; frankly, I get tired.”* Another participant (Ozan, Engineer, 31 yo, M, lives in Istanbul province) quoted, *“One does not want to go out under these circumstances.*”

Nonetheless, as per participants, car ownership offers not only practical advantages in terms of transportation but also psychological and emotional benefits associated with empowerment and convenience. Ayşegül (43 yo, Business Owner, F, lives in Erzurum province) commented,

“*There is a significant difference between before and after I have my own car.” If you do not have a car, you are going places by taxi. For a woman, having her own car is a wonderful thing. It’s great to have that key in your pocket.*”

Furthermore, internet access played a vital role in leisure engagement to be aware of nearby activities. As Ozan (Engineer, 31 yo, M, lives in Istanbul province) stated, *“Social media is very effective in activities. You can get notified of activities.”*

In conclusion, the participants highlighted the complexity of accessibility in leisure. While province conveniences and transport alternatives might improve access to leisure time, difficulties in urban environments may reduce leisure time, and these can change between bigger cities and smaller cities in terms of sociocultural and physical facilities. Internet connection, particularly through social media, is crucial for remaining connected to leisure options and activities, and car ownership is regarded as a practical and powerful answer. These elements work together to make leisure activities more accessible in urban and city settings in Turkey.

3.e. Opportunities

This subtheme explains the unique difficulties and constraints that people residing in smaller cities confront in terms of leisure activities. Living in a smaller province presents challenges, particularly in terms of limited social opportunities and a lack of activity variety. Participants expressed a desire for more diverse experiences but were aware of practical and societal barriers that hindered such pursuits. Erhan (Nurse, 28 yo, M, lives in a small county of Manisa province) highlighted the limitations of living in a small county with a population of 100,000, stating, *“A town with a population of 100.000 is small. Social opportunities are limited. That’s why I can’t leave the house much.*”

Consistently, another participant who studied in North Cyprus and currently lives in Aksaray province stated,

“*It is very different because of the city I live in. For example, when I was in North Cyprus, everything was different at night. Now that I leave work at 6 p.m., it’s eight until I say, come home and eat. What can I do after 8 p.m.? It’s a small city, after all.*”

Dilara (26 yo, Psychologist, F)

Additionally, weather conditions, especially harsh winters, significantly influence human behaviour and leisure activities. The changing seasons have practical and psychological effects; as Gökçe (35 yo, Lecturer, F, who lives in Istanbul province) quoted, *“Cold and winter mean preventing many things (in Turkish culture).”*

Mahmut (33 yo, Guardian, M, lives in Van province) added, “*There is the issue of summer and winter. There are no winter/summer time changes due to the government’s decision. We leave the house before sunrise and enter at sunset.”*

In conclusion, people in smaller cities have unique obstacles due to the lack of sociocultural (e.g., theatres, cinema, concerts) and physical (e.g., indoor facilities) opportunities, the smaller population, and the influence of the weather on recreational activities. In smaller urban contexts, these characteristics can have a big impact on the kinds of leisure activities that are offered and how people enjoy their leisure time.

3.f. Roles and responsibilities

This subtheme sheds light on the complexity of the parental role, particularly for married individuals with kids. These people must delicately create a balance between putting their children’s needs first and juggling the demands of balancing employment and personal interests. Participants who were married and had children highlighted the intricate and multifaceted characteristics inherent in the parental role doing their occupations as co-occupations, wherein individuals frequently prioritise the well-being of their offspring while concurrently dealing with the demands of achieving harmony between their work and personal pursuits. The participants quoted,

“*Of course, there are obstacles, especially because I have a problem allocating time…I remember feeling very good when I was able to have me time, and sometimes I miss it. You know, it’s good to get married, but there is also a reverse side to getting married: you have to transfer your leisure time to the family. I spend time with my family. Since our child is younger, we cannot participate in many social activities.*”

(Ahmet, 31 yo, M, Engineer, lives in Düzce province)

“*Because my wife is taking care of our young children, she cannot participate much in her leisure time… In the evenings, we-as parents- prepare meals for the children, play games, and help with their homework. Responsibilities continues after work.*”

(Orhan, 38 yo, Teacher, M, lives in Muş province)

In conclusion, these findings highlight the complex and diverse nature of the parental position, where people must negotiate the difficulties of juggling the demands of different occupational roles from work, family, and personal time. Parenthood may provide happiness and fulfilment, but it also necessitates significant sacrifices and a change in priorities, especially for parents of little children. In Turkish culture, there are some restrictions for social events from the cultural perspective. Little children are not taken everywhere due to concerns regarding their sleep schedule and the possibility of their presence disturbing other attendees. These findings provide us with a clearer comprehension of how parental duties might affect people’s leisure activities and choices.

4. Well-being

In this theme, participants conveyed their emotional experiences associated with engaging in leisure activities. It investigates the effects of leisure activities on people’s general emotional health and offers insights into the emotional components of leisure.

4.a. Positive feelings

Positive feelings are emotional states that are characterised by positivity, contentment, and delight. They are often used in the context of leisure and well-being. Participants highlighted how their leisure activities give them joy, pleasure, and a sense of pride as they voiced positive feelings related to these hobbies. Some participants expressed that they have positive feelings associated with leisure participation; participants 4 and 6 stated that they feel happy to have leisure time and quoted,

“*I feel very good about doing something for myself. I listen to myself… I am very happy with the value that I give to myself… I think it gives you a lot of pleasure. It’s like happiness and pride combined.*”

(Dilara, 26 yo, F, Psychologist, lives in Aksaray province)

*“I feel the pleasure of this happiness*.”

(Seda, 27 yo, F, Teacher, lives in Hakkari province)

These quotes show that these people experience joy, fulfilment, and pride from their leisure activities, which is beneficial for their emotional health. These satisfying feelings are necessary for well-being and support a healthy and purposeful life.

4.b. Satisfaction

The subtheme explains that taking part in leisure activities makes individuals feel satisfied. The quotations from the participants emphasise how different sentiments and attitudes are used to communicate this satisfaction. The participants quoted,

*“It is as if I gave a gift to myself.”*

(Güneş, 31 yo, Lecturer, lives in Istanbul province)

“*Leisure is legendary for me. I’ve never been ahead of it; I’ve always tried to do it, but I wasn’t upset when I couldn’t. If I couldn’t today, let today pass; I’ll do it tomorrow. I say that this is how it should be, and I say that there is good in it.I am happy. Isn’t that the purpose of life?*”

(Şahin, Basketball Coach, 34 yo, lives in Kilis province)

Overall, participants respected the positive effects leisure has on their overall well-being and viewed it as a source of personal fulfilment. This fulfilment gave their leisure activities more depth and significance, supporting the notion that leisure is a necessary element of a fulfilling existence.

4.c. Resilience

Resilience is the ability to adapt, recover, and go on after facing difficulty, stress, or difficult circumstances. It is the capacity to endure and successfully deal with the challenges, setbacks, and painful events of life while maintaining one’s mental and emotional health. This study found a relationship between engaging in leisure activities and one’s capacity to handle challenging circumstances in life. As per the participants, their ability to cope with difficult situations in life increased by participating in leisure activities. Fatma (41 yo, F, Government Official, lives in Mersin Province), quoted,

“*Leisure keeps me motivated. Life is what drives me. You live in a world of ups and downs. Let me tell you, I love to be happy.*”

Neriman (44 yo, Government Official, F, lives in Istanbul province) emphasised how engaging in leisure-time activities contributed to an enhancement in her mental resilience, as she articulated, *“It brings me peace. Without them (at my leisure), I would think of heavier things. I think about things that I can lift in terms of structure and mind—very heavy things. But when I deal with that kind of stuff at my leisure, I never have such heavy thoughts.”*

In contrast, another participant expressed his inability to partake in leisure activities and the resulting occasional instability. He shared, *“I am shrinking; I feel very suffocated. I feel sick. When a person can’t spare time for himself, this is reflected on his face and also on his body language. I think people have mental problems when they can’t take time for themselves.* (Zehra, 42 yo, Business Owner, lives in Çorum province)

To summarise, all the participants stated that engaging in leisure activities improved their well-being; whether physically or mentally for some participants, leisure is a source of motivation and well-being, whereas a lack of leisure can cause discomfort and possibly serious mental health problems. This emphasises the value of leisure in fostering emotional health and mental toughness in the face of challenges in life.

5. Occupational Justice

5.a. Negative Emotions Associated with Inability to Participate in Leisure Time

The absence of engagement in leisure activities, coupled with a sense of incompleteness in individuals’ lives, causes negative emotions. Participants underlined the significance of planning leisure activities and the psychological impacts of unmet expectations, as quoted,

“*When I can’t do something or when I can’t do something with my friends, I get restless. I’m concentrating on doing that job. It gives me uneasiness. Because doing it gives me peace of mind. It gives me restlessness and unhappiness when I can’t do it…I am losing my mood; my energy is low.*”

(Gökçe, 35 yo, Lecturer, F, lives in Istanbul province)

Zehra (42 yo, Business Owner, lives in Çorum province), in addition, mentioned that being unable to partake in leisure activities made them feel uncomfortable and unhappy. The participant talked about feeling like they are falling behind and getting depressed when she does not take time for herself. As the participant quoted,

“*For example, that day, I get very uneasy if I can’t read the Holy Quran first… But when I don’t read books, I am very angry with myself. I say you left yourself behind, Zehra, again…I think I left myself behind. I feel very sad. For example, I think that I don’t take time for myself when I go for a walk. Again, I say you ignored yourself, Zehra.*”

Moreover, Arif (40 yo, Security Officer, M, lives in Osmaniye province) presented the concept of dual boredom, which includes feeling as though one is wasting time and not engaging in any activity and quoted,

“*Of course, when you can’t participate to your leisure time there is boredom, both because you can’t do it and because your time is wasted*”

5.b. Occupational disruption, deprivation, alienation, and imbalance

In this study, occupational disruption refers to the situation where persons are unable to participate in leisure activities due to unexpected events like financial inabilities, other responsibilities, or rapid life disruptions and has significant emotional and psychological consequences. This condition, known as occupational deprivation, is characterised by feelings of loss and frustration. Long-term, sustained occupational deprivation can lead to occupational imbalance, characterised by an inadequate distribution of activities in a person’s daily life.

Gülay (45 yo, F, Freelancer, lives in Bilecik province) portrayed this sense of occupational disruption and occupational deprivation, stating, *“When I can’t do my leisure activities, I feel like something is missing.”* Her story highlights the way the inability to participate in leisure activities leads to a stronger feeling of occupational alienation, where one feels alienated from areas of life that bring happiness and significance.

Ayşe (39 yo, Teacher, F, lives in Adıyaman province), stressed that there is generally a sense of deprivation and restlessness when one cannot have personal time and quoted,

“*When I can’t meet my friends, I sometimes feel good, but generally I feel incomplete. I feel restless when I don’t have time for myself. Even if I am not so tired, I still feel like I am not fully mentally rested.”*

One participant described feeling stagnant and waiting for something to happen, using the metaphor of being “pickled” and feeling sour. They expressed a desire for more in life and compared their current situation to a “Nuri Bilge Ceylan movie.” This likely refers to the slow and contemplative style of the filmmaker’s work, suggesting that the author felt their current situation is reminiscent of such a film. As quoted,

“*When I think the times, I cannot able to do my leisure…For instance, I think about myself in quarantine for COVID disease. I stand like this and wait to be picked. I am sour. The front of my house is open and has a view of a field). I looked straight ahead. But it’s meaningless. I probably wouldn’t want my whole life to be like this. It’s like a Nuri Bilge Ceylan movie.*”

(Özge, 30 yo, F, Research Assistant, lives in Ankara province)

In conclusion, this subtheme emphasises the significant influence of leisure and the effects of its absence on the physical and mental health of working individuals. Lack of participation opportunities (occupational disruption) can cause emotional anguish, feelings of unfulfillment and stagnation (occupational alienation and deprivation), and isolation, which emphasises the need for leisure time to foster well-being and mitigate occupational imbalance.

6. Flow of life
6.a. Activity preferences and 6.b. Experiences

This study’s participants admitted that the concept of leisure is dynamic and changes with various life phases and opportunities for personal development. This theme explains that leisure is not stable or constant throughout a person’s life; it alters and changes in reaction to different factors. Erhan (28 yo, Nurse, M, lives in small county of Manisa province) elaborated on how personal growth and experiences shape leisure choices. Highlighting how the concept of leisure evolves with the transition of life, emphasising its dynamic nature, he quoted,

“*Progression, for instance, modifies a number of your behaviours. Habits that you once enjoyed may now seem absurd, or you may now be able to appreciate activities you once considered impossible. In the end, man is a constantly evolving organism. Our beliefs, health, and mental state are all evolving. There are many factors that contribute to change. There are internal factors. All external factors have an effect. Even a person’s negative experiences influence every aspect of his life. At that time, I was hanging out with friends more, doing things like hiking and going out. Much rarer now. Maybe it’s because of age; it could be because everyone got married. We started to work. I don’t want it too much anymore; it’s more attractive to stay at home. My habits have changed. The pandemic has changed our habits a lot.*”

Nonetheless, Ayse (39 yo, Teacher, F, lives in Adıyaman province) acknowledged the changing nature of pleasure derived from leisure activities over time. They recalled, “*When I was a student, it was very enjoyable to sit and chat with my friends in cafes, go to folk song cafes, and go to places with live music. But over the years, you realise that those activities don’t give you the same pleasure anymore.”*

Participant 18 (40, M, Security Officer, lives in Osmaniye province) likewise reflected on how age and life circumstances have altered their leisure choices and explained, “*Of course, ten years ago, there were fewer children; we were younger. We could get off the night shift and go on a picnic without sleeping. Now, as we get older, we can’t do these things; they don’t happen without rest. We used to play rummicub (light smile), where two or three families would come together and play card games. We’ve given them a break for three or five years now. “*

To conclude, the participants argued that leisure is not a static idea, but rather one that adapts and changes along with their own development, experiences, life stages, and shifting preferences and finances. This viewpoint emphasises the significance of adaptability and flexibility in locating and taking pleasure in worthwhile leisure pursuits throughout one’s lifetime.

## 4. Discussion

The primary objective of this study was to examine the importance and experiences associated with leisure-time participation among individuals who are Turkish working adults. The aim was to gain a comprehensive understanding of the meaning of participation and its overall connection throughout the process of participation. The findings of this study indicate that leisure participation exhibits a dynamic characteristic and can change throughout adulthood, with the very meaning of leisure being an essential aspect and the factor of the flow of life exerting the most significant influence on participation. Understanding the underlying causes, including environmental influences, is crucial to interpreting these findings. The recognition of the importance of leisure activities in fostering flow experiences contributes to an in-depth understanding of how meaningful participation can have a beneficial influence on individuals’ lives. Drawing a picture of the participation of working adults holds significant importance in terms of informing policies and practices that promote well-being, occupational justice, work–life balance, and overall life satisfaction in working adults.

### 4.1. Leisure Definition

This study found that Turkish people used “free time” and “leisure time” interchangeably but assigned different meanings to them distinguishing between idle/empty free time versus leisure time when they are engaged in pleasurable, fulfilling activities with meaning. Leisure time was when activities could be freely chosen as contrasted with obligatory activities. Leisure was seen as restorative, enabling “me time” and providing social support and recovery from work. Demirbas addressed [52] shifting away from the conventional definition of “free time activities” as the definition of leisure, which is still widespread in many languages around the world and focused on language-based hurdles in leisure studies, especially in Turkish. The first objective of the present study was to establish a shared understanding of the term “leisure” among the participants. Participants in the context of Turkish culture strongly differentiated leisure from free time by their definition, which is a direct translation and common understanding of “leisure” into the Turkish language and recalling idle time. These activities ranged from having some time just for a cup of tea or coffee to coffee meetings with friends for both genders. The remarkable point of view on leisure was that leisure time could be anytime and was supposed to be meaningful to them, not filled with idle things such as sleeping or scrolling social media. These results showed that working adults in Turkey participated in their leisure time based on their definition, and this incorporation of leisure activities embedded their daily lives with diverse meanings and was far away from solely unoccupied time pursuits, as Demirbaş addressed in 2022.

### 4.2. Meaning of Leisure

In the present study, the “me time” concept can shape leisure, which was defined by the participants as freely chosen and planned activities that bring pleasure and joy and leave you feeling rested and relaxed [53]. In addition, Rowe [54] describes what is perceived as leisure as the complexity that constantly reshapes and builds its meaning. While individuals who place leisure time at the centre of everyday life recognise this complexity, it is regarded as a key area of life that enables people to create meaning for the quest for a purposeful, enriched, and satisfying life by providing people with opportunities for active engagement with themselves, with others and with nature/the world. Based on this study’s findings, regardless of the type of leisure activity pursued and its level of importance to individuals, leisure assumes a fundamental role in their lives beyond the age of 30. It is noteworthy that those who are under the age of 33 demonstrate a notable lack of awareness of the fundamental notion of leisure. According to the perspectives expressed by participants, the notion of leisure might assume a fundamental role when individuals have achieved a certain level of stability in their lives. According to Havinghurst’s early adulthood developmental tasks, young adults in the later stages of their development tend to prioritise life transitions like marriage, employment, and career advancement over leisure activities [55]. This mostly concerns the cultural background of Turkey, where the culture emphasises economic independence and where significant life milestones such as the completion of higher education, attainment of a job, initiation of marriage, establishment of a family, and acquisition of a car and house have substantial importance [56]. It is imperative to consider that the findings demonstrate the prevailing lifestyle preferences among persons who come from a middle-class socioeconomic background, possess a college education, and belong to generation Y. The reason might be Havinghurst’s early adulthood—joining the workforce and starting a career, founding a family—has moved to the late twenties, and individuals feel a crunch between work and their lives, and work–life balance (or occupational balance) may not be as well established in early adulthood as it is in middle adulthood [20].

### 4.3. Flow of the Life

Alongside these developmental stages, the flow of life has played a role in leisure pursuits and how it could be realised in someone’s life. This research found that social surroundings, employment environments, working circumstances, extra responsibilities such as being a partner/spouse or parent, or changing tastes might influence someone’s leisure interests and preferences, as well as leisure participation.

“*Habits that you once enjoyed may now seem absurd, or you may now be able to appreciate activities you once considered impossible. In the end, man is a constantly evolving organism*”

(Erhan)

The quote is an excellent summary of someone’s changing life. It is important to note that this also reveals a bidirectional and dynamic relationship between the meaning of leisure experiences and leisure activity preferences. Flow is the essential core element in this dynamic relationship; it could be affected by the circumstances themselves or affect the meaning of leisure itself. Since the dynamism of flow has an overall effect on leisure participation, pursuits, well-being, and occupational injustice, from this point of the discussion, the flow of life and the dynamism of the flow will be discussed in harmony with the other themes in the next passages.

### 4.4. Facilitators–Barriers

During the interviews, participants were asked what their facilitators and limitations could be; it is captivating to note that originating from the flow of life, the facilitators can become limiters with time, and vice versa; furthermore, one limitation for an individual could serve as a facilitator element for another. The topic of participation limitations, particularly in terms of leisure participation, and its repercussions are frequently discussed in relation to individuals with disabilities, children, and the elderly. However, it is important to note that these limitations are sometimes overlooked and not readily apparent in the context of working adults. The findings of the present study explored the role of working conditions, more particularly flexible working hours, in enabling individuals to partake in leisure pursuits. These findings align with the findings of Kim and Cho, who highlighted that the majority of employed persons do not participate in leisure-based physical activities [57]. When examining gender differences, findings indicated that male workers with non-shift, overtime, precarious, and manual roles were less likely to engage in leisure activities, while positions such as manual labour and labour time and overtime-only positions influenced low levels of leisure participation. Moreover, the present study indicated the accessibility and availability of opportunities and financial resources as significant facilitators for leisure participation. In the context of Turkey, Gürbüz and Henderson illustrated those inadequate facilities, challenges in reaching opportunities, and limited financial resources as key constraints to leisure participation [58]. In line with the present study findings, evidence demonstrates social support as a potential source of this behaviour. For instance, a number of cross-sectional studies have suggested a significant association between social support and increased physical activity participation [59,60,61]. These factors include the presence of support from people and places, the availability of opportunities for socialisation, flexibility, the presence of inclusive leaders, and the presence of support to enhance a person’s sense of preparedness. Similarly, according to the findings of White et al., the facilitators associated with leisure participation include self-management and self-care, finding meaning, social support and networks, welcoming recreational settings, and being informed and educating others [62].

As inflation and the cost of living in Turkey persistently increase [63], individuals implement a reduction in their discretionary costs. This reduction is primarily directed towards prioritising essential needs such as rent payments and grocery shopping rather than indulging in dining out for dinner or socialising over coffee and dessert. This behavioural shift can be directly linked to the fundamental levels of Maslow’s hierarchy of needs, and financial satisfaction directly affects leisure satisfaction [64,65]. The aforementioned findings suggest that socioeconomic fluctuations exert an influence on leisure preferences within the context of Turkey. Arora et al. [66] report that Turkish consumers are mostly anxious about their health, the economy, and uncertainty. Additionally, a relatively recent survey conducted in the United Kingdom reveals that the escalating cost of living has resulted in a decline in leisure pursuits, compelling individuals to confront challenging decisions [67]. This underscores the global nature of the issue at present. When considering the process of saving money, individuals often prioritise reducing their expenditures in the realm of leisure activities. On the other hand, results reveal that participants who believed that their salaries were inadequate due to the escalating economic challenges in Turkey over the past few years and encountered difficulties in supporting their families expressed their commitment to utilising their free time to acquire language skills. Their objective was to enhance their professional prospects and seek opportunities abroad, with the aim of attaining improved living standards. Thus, they were using their non-work hours and allocating their time to learn a new language to migrate to another country.

“*I am a nurse. I want to learn the language by myself. I study German in my spare time, usually. It takes most of my time. I want to work abroad and practise my profession there. I have a purpose.”*

(Erhan)

The findings are consistent with the notion that individuals, upon recognising the disparities in living conditions and opportunities, may experience not only dissatisfaction but also, in cases of heightened desperation, a motivation to seek better prospects by migrating to countries that offer significantly improved quality of life [68].

### 4.5. Recovery and Well-Being

Based on the preferences for leisure pursuits, the participants expressed comparable improvements in terms of relaxation, mastery, and detachment from work. In relation to the concept of leisure, the majority of participants expressed that it encompasses a period of time dedicated to relaxation, which is the most commonly addressed dimension of leisure as in the Kuykendall et al. study [69]. Leisure gives the participants time to escape from the hustle and bustle of their lives in the present study. At first sight, this “escape” word may lead to an interpretation of ignoring the problems [70]. Alternatively, we might consider these findings derived from the participants’ declarations of experiencing a state of relaxation and dedicating time to themselves (me time). This form of relaxation has been shown to enhance mental resilience and shield individuals from emotional challenges, providing them with the necessary strength to cope with the various challenges of life, and this aligns with [71]. According to the results of the present study, leisure participation increases subjective well-being through resilience. The key point here for leisure participation is the meaning itself, based on the individual’s needs. As indicated, *“Leisure It is as if I gave a gift to myself.” (Güneş)* Evidence demonstrates that engaging in leisure activities increases the levels of resilience, subsequently diminishing psychological problems. Thus, leisure participation in Turkish community acts as a buffer against stressful experiences by fostering positive emotions associated with personal fulfilment and well-being, as suggested by the findings of Tagukichi et all [72].

However, it is important to note that for some participants, this relaxation is expected to encompass both physical and mental aspects, for which we use the broad term of psychological detachment from work in the present study, as Sonnentag and Fritz [73] describe. Notably, individuals in the present study have played a key role in this exploration. The participants’ definition is consistent with the idea of psychological detachment from the work of Sonnentag and Fritz, defined as abstaining from work-related thoughts and attaining mental distance from work during non-working hours. Similar to this, working individuals who participate in leisure activities that satisfy their desire for detachment and relaxation may see the biggest improvements in subjective well-being [3].

One of the main findings of this study is participation in leisure activities increases an individual’s resilience and provides them with a sense of purpose in life. The findings demonstrate leisure as a salient source of well-being among Turkish working adults in different ways. In line with these, a longitudinal population-based study among middle-aged adults has demonstrated a significant role of increasing levels of leisure engagement in improving health and well-being [18]. It is evident that individuals who are more committed to leisure activities are more likely to experience satisfaction, which is linked to overall happiness, and the social aspects of leisure satisfaction predict happiness in long-term [74]. Similar to current study findings, evidence demonstrates that leisure engagement improves emotional well-being and helps in accumulating psychological resources such as social support and optimism, which in turn counteract stressors [75]. Likewise, another study suggests a significant relationship between leisure coping and positive affect, mitigating perceived stress and promoting overall well-being [71]. This is consistent with Fredrickson’s Broaden-and-Build theory [76], indicating a thought–action repertoire that builds psychological resources over time. Thus, leisure participation assists individuals in developing psychological resources via a Broaden-and-Build mechanism, causing higher levels of well-being and buffering against stress over time. Young adults develop occupational resilience by being able to adapt to adverse circumstances and continuously engaging in meaningful occupations such as leisure [77].

Furthermore, participants in this study exhibit a preference for devoting their free time to developing mastery in a certain area, such as earning mastery in playing the guitar or committing time to improving their skills in work-related tasks. The expression of the interpretation of leisure and work time is discussed in relation to the idea of dedicating leisure time to job-related duties. The notion of work-related mastery time can be examined in relation to incomplete psychological detachment, individuals’ affinity for their job, and their increased sense of security when they participate in self-improvement activities such as reading or attending work-related courses [78]. Likewise, it is illustrated that participating in leisure pursuits that demand expertise and understanding distinct from those required in one’s profession improves job performance effectiveness alone when leisure activities are approached with a committed and significant attitude [79,80].

### 4.6. Occupational Injustice

One of the main findings of the present study is that working adults of working age encounter occupational injustice in different layers within all aspects of occupational disruption, occupational deprivation, occupational alienation, and occupational imbalance even though they do not have any disabilities or illness. Occupational injustices occur from time to time in the flow of life, as unsupporting consequences of participation limitations among working adults. These disruptions in the balance of daily activities underscore the importance of leisure for maintaining overall health and well-being. One can shift from well-being to a lack of well-being in the dynamic flow of life, which leads to occupational injustice over time. These results draw excessive attention to the fact that working adults can face occupational injustice; even though occupational justice is mostly discussed in the literature for vulnerable people who have immigration histories [81] or people with disabilities [82]. From an occupational science and occupational justice standpoint, every individual has the right to engage in any desired occupation [83]. When individuals encounter a lack of engagement or are unable to engage in leisure activities according to their preferences and wishes, the initial occurrence is occupational disruption as a consequence of the participation limitations stated by participants in this study. As limitations are stated in this study as other roles, e.g., responsibilities, being a parent or spouse, occupational disruption could be considered regarding the re-organisation of their lives by establishing several priorities [84]. Similarly, participants report experiencing a great deal of occupational disruption, occupational deprivation, and occupational imbalance when they are unable to participate in their leisure activities, including the period of the COVID-19 pandemic [77], thus leading to the perception of negative emotions such as uneasiness and unhappiness. Later, the phenomenon is known as occupational deprivation and serves as a precursor to subsequent features, such as feeling like there is something missing in their lives. The participants in the present study convey their personal experiences, drawing upon Crabtree’s examination of occupational deprivation within the prison context [85] which refers to ‘dead time’, gradual erosion, and loss of meaning. This phenomenon arises from a diminished engagement in leisure pursuits and a subjective perception of an unfulfilled aspect of one’s overall lifestyle. As the gap widens between the desire to participate in meaningful leisure occupations and the actual frequency and quality of participation, the consequences manifest as occupational alienation and occupational imbalance. This study underscores the importance of addressing these injustices to enhance the well-being and quality of life of working adults.

## 5. Conclusions

This study concludes that while Turkish people use “free time” and “leisure time” interchangeably, they distinguish between idle time and meaningful leisure that offers personal fulfilment, autonomy, and recovery from work. Moreover, this study shows that people can ascribe meaning to any kind of leisure activity, and any leisure activity that individuals find meaningful can contribute to occupational balance and resilience. In Turkish culture, leisure becomes particularly significant in a person’s mid-thirties, a time when adults are balancing the demands of their careers and personal lives while navigating the complexities of adulthood within the dynamic flow. The findings suggest that leisure is not just a form of relaxation but is closely tied to broader environmental, economic, and social factors, influencing overall well-being. The findings indicate that working adults may experience limitations to their leisure participation and varying degrees of occupational injustice over time, even though they do not have any health problem. By recognizing the importance of leisure, especially during adulthood, individuals can find meaning in their daily occupations, leading to a more balanced and fulfilling life. By acknowledging and enhancing leisure participation, individuals can achieve a more balanced and fulfilling life, thereby reducing occupational injustice and promoting overall well-being and occupational justice. Although there are some limitations in this study, such as the fact that the study’s sample may not capture the full range of experiences across different region, socio-economic classes, and the vibrant culturally mosaic backgrounds in Turkey. Moreover, the study’s cross-sectional design limits our ability to observe changes in leisure perceptions and practices over time. Additionally, while the findings are pertinent to Turkish culture, and they may not be generalizable to other cultural contexts with different leisure values.

## 6. Implications 

The implications of this study suggest that therapists should integrate leisure activities into their interventions to promote occupational balance and well-being, as leisure plays a crucial role in working adults’ lives, particularly in Turkish culture. Policymakers are encouraged to improve macro external factors such as work–life balance policies, infrastructure, and access to leisure opportunities to support occupational justice. Furthermore, it is important to take into account culturally sensitive strategies while implementing leisure intervention, as the preferences and interests of middle-aged adults may change over time. Future research could examine the differences in leisure engagement between Turkish working adults and individuals from diverse cultural backgrounds in order to understand how culture influences leisure preferences, interpretations, and obstacles. Additionally, future research could explore how the changes in leisure patterns over time in Turkish culture and across cultures inform the development of culturally responsive policies and practices.

## Figures and Tables

**Table 1:** Semi-structured interview questions [based on [3] (pp. 633–658)].

Semi-Structured Interview Questions	Dimensions of Leisure Occupational profile [2].
Is the concept of free time more suitable for you than the leisure concept?	Sense-making, personal meaning
1. What does leisure mean to you?	Subjective experience and personal meaning
2. What comes to mind when you think of leisure?	Subjective experience and personal meaning
3. What do you do in your leisure time? 4. What did you do in the past? Are you still continuing? 5. What would you like to do in the future?	Activity preferences, temporal dimension
6. What times do you do it? (Summer, winter, seasonal features?)	Temporal dimension
7. For how long and how often do you do it?	Activity preferences
8. With whom would you prefer to do it?	Activity preferences
9. What does participate in leisure mean to you?	Subjective experience, and satisfaction from experience
10. How does it make you feel to participate in leisure time? How do you feel when you can’t attend?	Subjective experience, and satisfaction from experience
11. What motivates you to do so? How do you feel when you can’t attend?	Subjective experience, and satisfaction from experience
12. Can you do your leisure activities the way you want?	Environmental context and activity contexts Subjective experiences, satisfaction from the experience
13. Are there any cases where your leisure is affected? If yes, what are the influencing factors? What are the barriers? What are facilitators?	Subjective experiences, environmental factors, and activity contexts

**Table 2:** Sociodemographic data of the participants.

		n
Gender	Female	14
	Male	14
Age	Min	25
	Max	50
	Average age	34
Education	2 years college	6
	4 years university (Bachelor’s degree)	15
	6 years university (Medicine)	1
	Master’ degree	5
	PhD	1
Living (with)	Single	5
	Homemate/s	2
	Spouse/partner	6
	Parents	2
	Family with kids	13
Monthly Income	1x or less	3
	1x–1.5x	5
	2x–3x	12
	3x–4x	4
	4x and above	4
Working style	Full-time	24
	Part-time	4
	Night shifts	5
	Hybrid (Home office and in office)	3
	Home office	2
Working hours (weekly)	0–20 h	3
	20–30 h	5
	40 h	7
	40–50 h	9
	50–60 h	1
	60–70 h	2
	70 h (with shifts)	1
	n = 28, x = Minimum wage in Turkey	- Minimum wage in Turkey - September 2021 USD 598 - January 2022 USD 483 - May 2022 USD 418

**Table 3:** Themes and subthemes of the study.

**Meaning of leisure**	Freedom Leisure instead of free time Me time
**Recovery from work**	Relaxation Mastery Detachment
**Facilitators and Barriers**	Working conditions Financial resources Accessibility Roles and responsibilities Social support systems Opportunities
**Wellbeing**	Positive emotions Satisfaction Resilience
**Occupational Injustice**	Negative emotions related to a lack of participation Occupational disruption Occupational deprivation Occupational alienation Occupational imbalance
**Flow of life**	Activity preferences Experiences

## Data Availability

All necessary data are available from the manuscript. The authors will share the available dataset if required.
